# One-size-fits-all or friend-specific? A social relations model approach to social support in friendships

**DOI:** 10.1177/02654075261435224

**Published:** 2026-03-16

**Authors:** Anas Alsayed Hasan, Sarra Jiwa, Victoria Pringle, Erika Nicole Carlson

**Affiliations:** 1 8637 University of Windsor, Canada; 2 University of Toronto, Canada

**Keywords:** Friendship, social support, individual differences

## Abstract

When providing support to a friend in need, do we tailor our support to the specific person and situation in front of us? Past work tends to assume that this is the case, but it’s possible that people have broader support-giving tendencies that persist regardless of the context. Here, we decomposed sources of social support using the Social Relations Model among close friend groups (*N* = 328 participants, 87 groups, 522 dyads). We found consistent evidence that social support is both a general tendency and a dynamic process, which destabilizes theorizing of social support as an entirely relational (i.e., tailored) process. Overall, our results highlight the importance of accounting for different sources of variance in interpersonal processes.

When a friend is feeling stressed, people offer their support. What determines the type of support someone offers? Past work suggests that people tailor their support to the person and situation (i.e., support is a relational dynamic). However, like other interpersonal behaviours, perhaps people have general tendencies to give and receive particular types of support as well. Given that past work focuses on dyadic interactions, which makes it impossible to tease apart relational dynamics from broader tendencies, we use the Social Relations Model (SRM; Kenny, 1994) in the current work to tease these possibilities apart in friend groups.

## Social support: individual difference or relationship dynamic?

Most commonly, support is divided into four types: *emotional support*, which reduces distress through empathy and reassurance; *informational support*, which involves problem-solving and advice-giving; *tangible support*, which refers to concrete favours; and *esteemed support*, which consists of compliments, validation, or praise. Notably, people can also engage in *negative behaviours* such as criticizing, blaming, or interrupting when attempting to provide support (Suhr et al., 2004). As for which type of support is best, some support behaviours are more effective in some situations and for some people more than others (Cutrona et al., 2007; Haven, 1994; Mikulincer & Florian, 1997). Thus, to provide effective support, people should be adjusting their support depending on the situation.

While past work has explored the types of support offered in certain contexts, here we explore the degree to which support provision is multidetermined by individual differences in how people *give* and/or *elicit* support, and the relational dynamics (tailoring). The Social Relations Model (SRM) provides a theoretical and statistical framework for understanding these different sources of social support.

In the SRM, *actor effects* refer to support-*giving* tendencies. For example, Paula provides emotional support to Rahul because she tends to use emotional support with everybody. Evidence for actor effects comes from one study which showed that when supporting the same friend across multiple days, people were consistent in whether they offered more emotional or tangible support (Morelli et al., 2015).

Another component of the SRM is *partner effects*, which refer to support-*eliciting* tendencies. For example, Paula gives emotional support to Rahul because Rahul tends to elicit emotional support from others in general, but perhaps she gives Ria less emotional support because Ria is uncomfortable with connection (e.g., she is more avoidant). Indirect evidence for partner effects comes from research on attachment styles, which shows that those with avoidant attachment tend to prefer instrumental support over emotional support (Girme et al., 2015; Mikulincer & Florian, 1997), and in turn, might elicit this style from others.

The final component of the SRM is *relationship effects*, which refer to a unique dyadic dynamic. Specifically, Paula provides more emotional support to Rahul because of their unique closeness and/or because Rahul’s current situation called for more support. Evidence for relationship effects comes from findings that relationships with greater depth tend to display more emotional support (Devoldre et al., 2010).

Using the SRM to understand how social support arises has several implications on social support research. First, it can advance our understanding of when and why support is effective (e.g., is success due to tailoring or due to the individual differences people bring with them?). Second, it can guide research on the predictors of support behaviour (i.e., characteristics of the provider, recipient, or their relationship) and outcomes (i.e., which supportive strategies foster better relationships, coping, or problem-solving). Finally, the SRM offers a more precise framework for exploring support behaviour by source (e.g., relationship effect of support x relationship characteristics) rather than conflating these sources of variance.

Notably, while the SRM has been applied to *perceived* social support, it has not been applied to *enacted* social support. A couple of studies have attempted to tease apart the social and trait influences of enacted support using other statistical approaches, though not without major limitations (Lakey et al., 2010; Woods et al., 2016). First, this work relied on retrospective self-reports, which are susceptible to an array of biases (Podsakoff et al., 2003) and can be distorted by the individual’s global attitudes towards the target (e.g., relationship liking; Joel et al., 2025). Second, this work did not differentiate between the *types* of social support (e.g., emotional, informational, esteemed), a practice criticized for obscuring the heterogeneity of support behaviour (Taylor, 2011). Lastly, the statistical approach did not account for actor effects (i.e., only recipient and social effects).

## Research overview

When people support their friends, to what extent does their behaviour reflect an individual difference in providing support, the other person’s tendency to elicit certain forms of support, or a relational dynamic uniquely tailored to the specific friend or problem? To date, most work has studied dyadic interactions in ways that make it impossible to tease apart these possibilities. As for the little research that has measured support across different relationships, none have done so using behavioural measures, a round-robin design (which allows for a full SRM decomposition), or classifications of different support behaviours.

In the current work, we behaviourally code dyadic support interactions in friend groups of four, which allows us to quantify the proportion of variance in support behaviour as explained by actor effects (support-giving tendencies), partner effects (support-eliciting tendencies), and relationship effects (relationship dynamics). In line with existing SRM benchmarks for interpersonal behaviours, we expect that relationship effects will account for most of the variance in enacted support, followed by actor effects, with partner effects contributing minimally (Back & Kenny, 2010; Kenny et al., 2001).

Our data comes from a larger study (see https://osf.io/7x8ab/overview) but has not been used to answer the questions examined herein. The materials and syntax for the present work are available at https://osf.io/9vpza/.

## Method

### Participants

Participants (*N* = 348; *M*_age_ = 20.56 years, *SD*_*age*_ = 5.88; 21.2% male, 72.1% female, 4.1% nonbinary, 2.6% self-identified) were recruited in friend groups of four (*N* = 87 groups, *n* = 522 dyads) from the University of Toronto Mississauga and the surrounding community. Participants were ethnically diverse (41.9% South/Southeast Asian, 16.3% East Asian, 11.6% Middle Eastern or North African, 11% White, 8.1% multiethnic, 6.4% Black, 3.2% self-identified, and 1.5% Latin American). Subjective socioeconomic status was assessed using a single-item ladder measure (1 = *bottom rung*, 10 = *top rung*), with participants reporting moderate standing on average (*M* = 5.74, *SD* = 1.92).

We excluded participants who reported that they didn’t know someone well in their group (e.g., if mutual friends nominated someone the other friend didn’t know well) or if coders indicated that participants did not stay on topic (*N*_excluded_ = 23), leaving a final sample of 325 participants. Participants were compensated with either a $30 Amazon gift card, course credit, or a combination of the two.

### Procedure

Each person in the group was emailed a 60-minute online survey, during which they listed 3 current stressors, among other ratings of themselves and their friends. Next, they took part in a 2-hour Zoom session, where they were assigned to breakout rooms to have one-on-one conversations about each person’s stressor. The facilitator in the room turned off their camera and microphone as the participants discussed.

Each interaction was 10 minutes. During the first 5 minutes, one person discussed one of their stressors, which the research assistant privately reminded them of via the chat, while the other person listened and provided (unprompted) support; then, the research assistant prompted the pair to switch roles for the remaining 5 minutes.

### Measures

#### Support behaviour

Research assistants (*n* = 7) coded each dyadic conversation for the extent to which (a) the interaction was characterized by five broad types of support (emotional, informational, esteemed, tangible, negative)^1^ using a 5-point Likert scale (1 = *Not at all*, 5 = *Extremely*), and (b) specific support behaviours from each support type were present (e.g., validating, complimenting) using a 5-point Likert scale (1 = *Strongly disagree*, 5 = *Strongly agree*). Intercoder agreement was adequate (Table 1).

**Table 1.** Descriptive statistics for all coded support behaviours.

Support behaviour	Mean	SD	ICC3k
Emotional support	3.05	0.72	.81
Esteemed support	3.02	0.81	.86
Informational support	2.66	0.91	.90
Tangible aid	1.16	0.41	.87
Negative behaviours	1.64	0.57	.81

#### Stressors

Each participant listed 3 current life stressors, which they knew they would discuss with their friends during video-recorded conversations. The stressors included concerns about academics (28%; “grades”), close relationships (21%; “family dynamics”), career (14%; “workplace hardships”), the future (12%; “uncertainty”), health (9%; “anxiety”), self-esteem (9%; “my appearance”), and finances (8%; “bills”) (see Supplemental Material for codebook).

### Analyses

To decompose support behaviours into SRM components (Kenny, 1994) we used the TripleR package (Schönbrodt et al., 2012) in R (version 4.4.2; R Core Team, 2024). Furthermore, we used latent SRM to tease apart relationship variance from error. The two indicators for each support type were the global ratings of the support category and a composite of the specific behaviour ratings within a category (see Supplemental Material for details). Notably, SRM effects that represent less than 10% of the total variance are not practically meaningful (Kenny, 2020), and as such, we do not consider effects below this magnitude to be meaningful.

## Results

**Figure 1.**
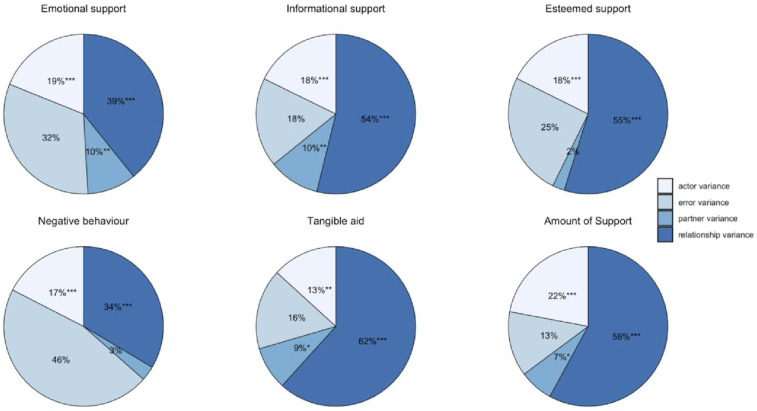
Social Relations Model Decomposition of Social Support. *Note*. * = *p* < .05, ** = p < .01, *** = *p* < .001.

Actor effects were significant for all types of support and for the overall amount of support, explaining 13–22% of the variance (Figure 1). Thus, people have consistent ways of providing support to their friends (e.g., part of why Paula provides emotional support to Rahul is her general tendency to give emotional support to all her friends).

Partner effects were only meaningful for emotional and informational support, explaining 10% of the variance in both cases (Figure 1). Thus, people reliably elicit emotional and informational support from others (e.g., part of why Paula provides emotional support to Rahul stems from Rahul’s tendency to elicit this style from his friends), but do not reliably elicit esteemed support, tangible aid, negative behaviours, or more/less support overall.

Relationship effects explained 34–62% of the variance in enacted support (Figure 1), suggesting that social support among friends largely, but not exclusively, reflects a relational process. Notably, although the specific proportions of actor, partner, relationship, and error variance differed across support-types, the relative contributions were the same, such that relationship variance explained most of the variance, followed by actor variance and then partner variance. Thus, part of Paula’s support-giving towards Rahul is due to her tendencies to give (and his tendencies to elicit) a particular type of support, but close to half of the variance in her behaviour is due to her unique response to Rahul and/or his problem.

## Discussion

Most work to date has assumed that social support involves adjusting support to fit the needs of the support recipient. While we found this strong relational component to support, we also observed robust stable components. Indeed, people had meaningful general tendencies to provide all five enacted support types, and to a lesser extent, people elicited some types of support. This suggests that a more complete account of support provision should consider both stable and relational components.

With respect to support-giving, we observed consistency in support provision across friends, but it is unclear if people have support styles that span different types of relationships (e.g., family, partners, coworkers). Either way, future work should identify traits associated with support giving (e.g., empathy) and any costs or benefits to these tendencies (e.g., relationship satisfaction).

With respect to reliably eliciting support, we only observed this effect for emotional and informational support. Ideally, future research will explore if this effect is observed across different relationships, who reliably elicits support, and if elicited support has positive or negative effects. For example, people who seem comfortable with intimacy (e.g., secure attachment) might elicit emotional support that feels satisfying, whereas people less comfortable with intimacy (e.g., attachment avoidance) might elicit informational support, which may or may not feel satisfying.

Finally, for all forms of enacted support, people seemed to tailor support to the recipient and/or stressor, which is consistent with existing theorizing that social support is a dynamic process (Cutrona et al., 2007). However, future work should employ the SRM approach to isolate the relational variables that explain why and/or how people alter their support behaviour to avoid confounding actor/partner effects. Notably, relationship variance was largest for tangible aid, suggesting that concrete favours are especially context-dependent, whereas it was smallest for negative behaviours, suggesting that unhelpful behaviours were more about the support-giver than the dynamic itself. Future work might explore the robustness of this pattern.

### Limitations and future directions

Despite methodological rigor (e.g., behavioural coding of support, round-robin design), this work has some limitations. First, we did not investigate variables that might predict actor, partner, or relationship effects (e.g., personality, attachment style, relationship quality). Future work that does so might explore how profiles of support-giving are associated with high-quality support (e.g., verbal person centeredness; High & Dillard, 2012). Related, our approach might be used to explain the discrepancy between *perceived* vs. *enacted* support (Lakey et al., 2010), by examining links among actor, partner, and relationship effects (e.g., do we see a stronger link between perceived and enacted support when correlating just the relationship effects of both?).

Second, most stressors were low in severity and were discussed in the lab. Future work might test if our effects replicate for more severe stressors (and/or when problems are more similar), as well as in naturalistic settings where conversations are less constrained.

## Supplemental material


Supplemental Material - One-size-fits-all or friend-specific? A social relations model approach to social support in friendships
Supplemental Material for One-size-fits-all or friend-specific? A social relations model approach to social support in friendships by Anas Alsayed Hasan, Sarra Jiwa, Victoria Pringle and Erika N. Carlson in Journal of Social and Personal Relationships

## Note

1. The support categories used for the coding scheme were based on the Social Support Behaviour Code (SSBC; Suhr et al., 2004).
