# Supplementary material for: One-size-fits-all or friend-specific? A social relations model approach to social support in friendships
Source: J Soc Pers Relat. 2026 Mar 16;43(9):2673–81. doi: 10.1177/02654075261435224 (PMC13436809; doi:10.1177/02654075261435224)
Supplement: Supplementary file 1 [file sj-pdf-1-spr-10.1177_02654075261435224.pdf]

Robustness Checks for Measures of Social Support

In order to disentangle relationship effects from error in the Social Relations Model, there must be two indicators of the overall construct. That is, in order to decompose emotional support, there needs to be two measures of emotional support. To get these two measures, we used the global ratings of each support type (e.g., coders' ratings of how much each support provider gave emotional support overall) and the average of the ratings of the lower-order behaviours in each support category (e.g., coders' ratings of how much each support providers' style was characterized by reassurance, empathy, sympathy and concern). Conceptually, these two measures should both be capturing the extent to which the provider gave emotional support, which is characterized by the lower order behaviours in the category. Therefore, this latent decomposition is the decomposition we report in the main text.

As a robustness check, to ensure that the results of the coding of the global ratings and the lower order behaviours were indeed measuring the same constructs (and therefore can be used as indicators of the same construct in the latent decomposition), we explored the degree to which coders' global ratings of support (e.g., emotional) correlated with their ratings of subscale behaviours (e.g., empathy, sympathy, reassurance, concern). The global rating for each category was strongly correlated with the composite ratings of its constituent behaviours: emotional support [r (965) = .73, $p < .001$], informational support [r (965) = .79, $p < .001$], esteemed support [r (965) = .77, $p < .001$], negative behaviour [r (965) = .70, $p < .001$], and tangible aid [r (965) = .84, $p < .001$]. This suggests that coders rated the general categories of support in a manner that was consistent with their observations of specific behaviours.

In addition, we ran a version of the SRM decomposition using only the global ratings of support and another using only the composites of support behaviours (Figure S1). While these

decompositions do not allow us to tease apart relationship variance from error (we only have a single indicator of support in each model), observing if the general pattern of the decomposition is similar regardless of which indicator we use serves as a robustness check that they both represent the same support type. Given that the relative patterns are similar across both decompositions using a single indicator, and the latent SRM decomposition, our use of both support measures as indicators of the same support type in the latent decomposition is appropriate.

Coding of Stressors

We coded the stressors into categories that included: academics (e.g., "grades"), close relationships (e.g., "family dynamics"), career (e.g., "workplace hardships"), the future (e.g., "future uncertainty"), health/mental health (e.g., "anxiety"), personal or self-esteem (e.g., "my appearance"), finances (e.g., "bills"), loss/grief (e.g., relative passed away) or other (e.g., worldly issues). The definitions for each of the categories were as follows:

- **Academic Pressure**: Stress related to schoolwork, exams, grades, and academic performance.
- **Career Pressure:** Stress related to job performance, career advancement, work responsibilities, and challenges in finding or advancing in a career path.
- **Financial Concerns:** Stress specifically related to money management, financial stability, and meeting financial obligations.
- **Family/Relationship Issues:** Stress from interactions with family members, romantic relationships, and friendships.
- **Health/Mental Health:** Stress related to physical health issues and mental health challenges.

- **Uncertainty About the Future:** Stress related to unpredictability and lack of clarity about upcoming life events, decisions, and general future planning.
- **Personal Development/Self-Esteem:** Stress related to self-worth, confidence, and striving to meet personal aspirations and improve oneself.
- **Loss/Grief:** Stress related to experiencing loss, such as the death of a loved one or the end of a significant relationship.
- **Other:** Stressors that do not fit neatly into the other categories, often relating to logistical, technical, or miscellaneous issues.

The de-identified stressors were first passed through AI (ChatGPT) to categorize the stressors into these categories. To ensure the coding was correct, 7 research assistants (different research assistants to those who did the social support coding) each looked at a subset of the stressors and indicated if they agreed with the classification. If they disagreed, they re-coded the stressor into the category they thought best fit the stressor, using the definitions above, and provided an explanation as to why they disagreed. Then, two authors from the research team independently looked through all stressors where the RA disagreed with the AI coding. For these instances, the authors made the final decision as to which category(ies) best captured the stressor. In some cases, stressors were coded into more than one category (e.g., "money (looking for a job)" would be coded into career pressure and financial concerns).

Figure S1

SRM Decomposition using global measures of support (a) and SRM Decomposition using composites of support behaviours (b)

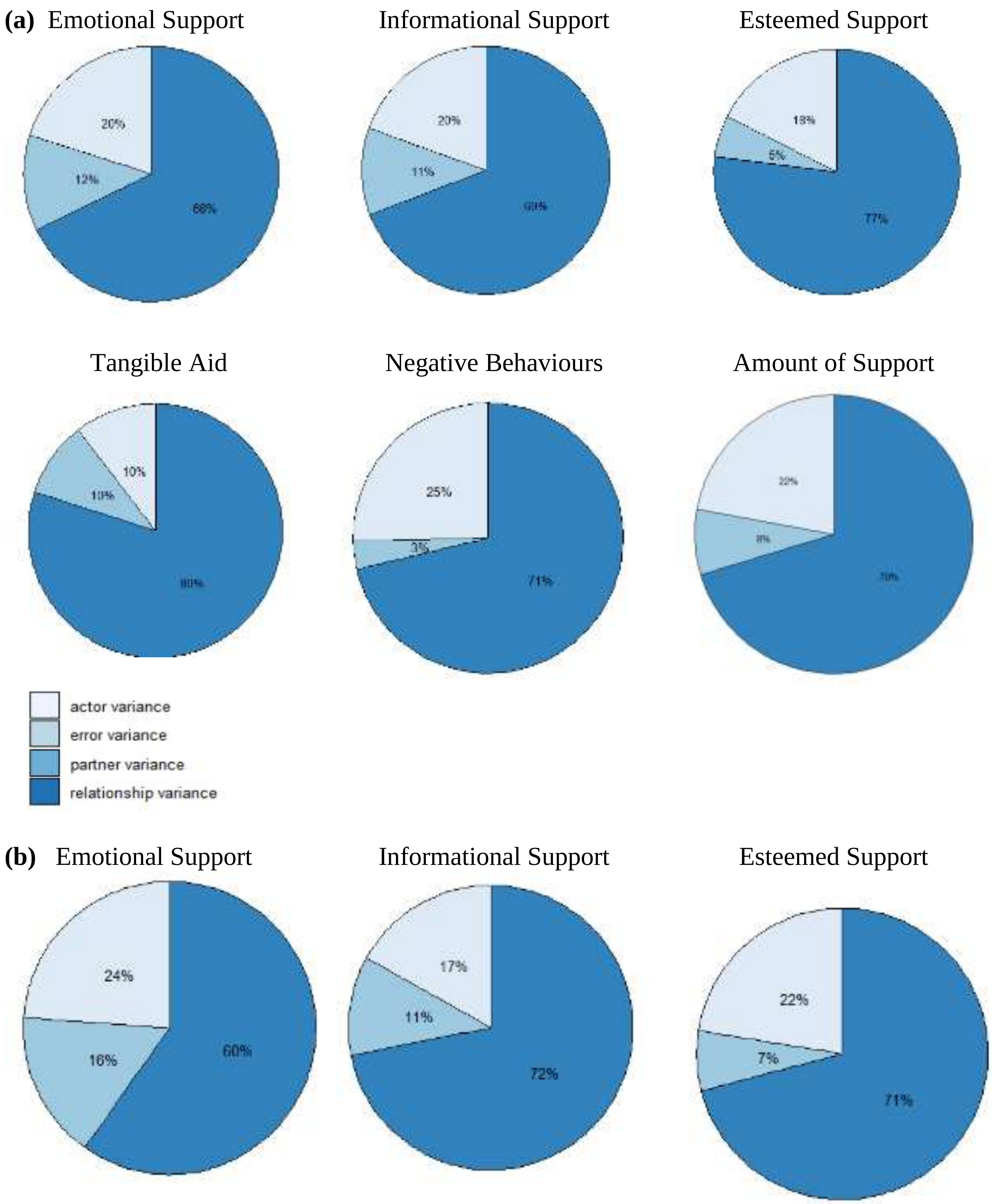

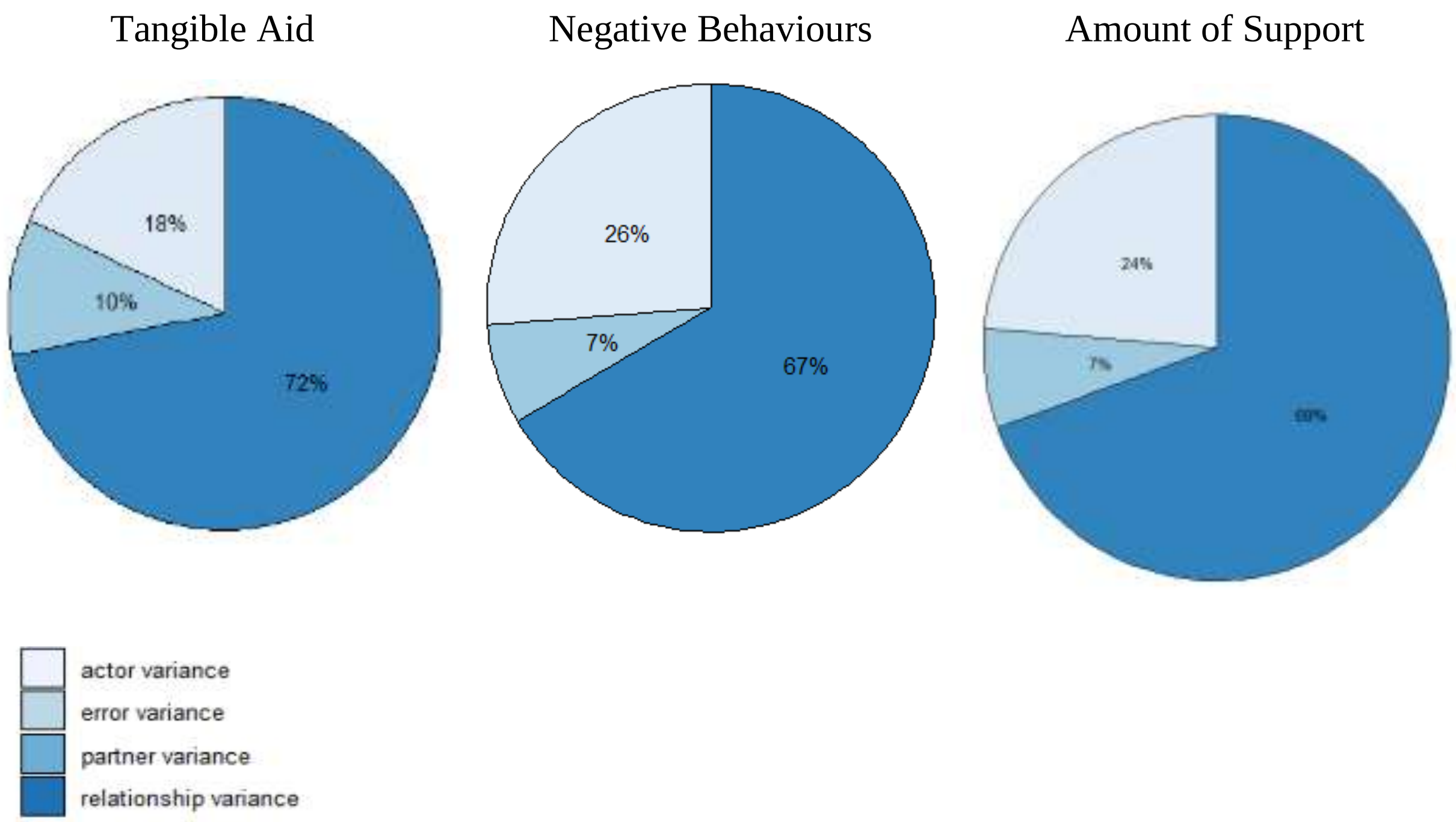
Tangible Aid
Negative Behaviours
Amount of Support
18%
10%
72%
26%
7%
67%
24%
7%
actor variance
error variance
partner variance
relationship variance
